# An observational study substantiating the statistical significance of cardiopulmonary exercise with laboratory tests during the acute and subacute phases of center and home-based cardiac rehabilitation

**DOI:** 10.1097/MD.0000000000026861

**Published:** 2021-08-06

**Authors:** Jeong Jae Lee, Jun Young Ko, Seungbok Lee

**Affiliations:** MJ Cardiac Rehabilitation Center, Department of Physical Medicine and Rehabilitation, Myongji Hospital, Goyang, Republic of Korea.

**Keywords:** cardiac rehabilitation, cardiovascular disease, center-based, CPX, home-based, laboratory tests

## Abstract

Cardiac rehabilitation (CR) can improve clinical indicators in patients with cardiovascular diseases. The literature reports a 20% reduction in all-cause mortality and a 27% reduction in heart-disease mortality following CR. Although its clinical efficacy has been established, there is uncertainty whether center-based (CBCR) is more effective than home-based (HBCR) programs in acute and subacute phases. We aimed to verify significant differences in their effectiveness for the improvement of cardiopulmonary function by analyzing cardiopulmonary exercise (CPX) with laboratory tests following both CR programs.

A single-center cohort study of 37 patients, recently diagnosed with underlying cardiovascular diseases, underwent CBCR(18) and HBCR(19). CBCR group performed a supervised exercise regimen at the CR center, for 1 hour, 2 to 3 days a week, for a total of 12 to18 weeks. HBCR group completed a self-monitored exercise program at home under the same guidelines as CBCR. Participants were evaluated by CPX with laboratory tests at 1- and 6-month, following the respective programs.

There was no statistical significance in clinical characteristics and laboratory findings. Pre–post treatment comparison showed significant improvement in VO2/kg, minute ventilation/carbon dioxide production slope, breathing reserve, tidal volume (VT), heart rate recovery, oxygen consumption per heart rate, low-density lipoprotein (LDL), LDL/HDL ratio, total cholesterol, ejection fraction (EF) (*P* < .05). CBCR approach showed greater improvement with significance in VO2/kg, metabolic equivalents, and EF on between groups analysis (*P* < .05).

The time effect of CPX test and laboratory data showed improvement in cardiopulmonary function and serum indicators for both groups. VO2/kg, metabolic equivalents, and EF were among the variables that showed significant differences between groups. In the acute and subacute phases of 1 to 6 months, the CBCR group showed a greater cardiac output improvement than the HBCR group.

## 1 Introduction

Cardiac rehabilitation (CR) is associated with many benefits, including improved quality of life, psychosocial well-being, and cardiorespiratory fitness.^[1]^ It is also known to improve modifiable clinical indicators, reduce known risk factors, and increase overall body function.^[2]^ The literature reports that patients with ischemic heart disease (IHD) receiving CR have a 20% and 27% reduction in all-cause and heart-disease-related mortalities, respectively.^[3,4]^ A Cochrane review of 147 studies demonstrated that, for myocardial infarction and heart failure, cardiovascular mortality and readmission rates were reduced by 25% and by 20%, respectively.^[5,6]^ A meta-analysis by Kabboul in 2018 compared the effects of core components of CR on morbidity and mortality, thereby showing a significant reduction in all-cause mortality.^[7]^

Despite current international guidelines promoted by the American Heart Association and the European Society of Cardiology, among others, CR is significantly at a global minimum with varying rates.^[8]^ The reasons include the lack of programs across centers worldwide and low referral rates compounded by transportation and cost barriers.^[9–12]^

However, these variables can be mitigated^[13]^ with clinicians and medical centers’ collaborative efforts by appropriately promoting CR to patients at the bedside.^[14]^

Traditionally CR programs have been medical center-based and conducted in an outpatient setting under medically trained and licensed specialists. Home-based cardiac rehabilitation (HBCR) was first introduced in North America and Italy. One study—a nonrandomized controlled quasi-experimental investigation—based out of Italy (Zaanelli, 2013) paved the path for standardizing underlying principles of a home-based approach to CR.^[15]^ The incentive for pursuing the study stemmed from the need for CR among cardiac postsurgical patients who lived in remote rural areas, where center-based (CBCR) programs were nonexistent. Since then, studies throughout the literature have similarly reported that HBCR showed improvement of relevant symptoms and clinically meaningful indicators.^[15]^

However, few studies have analyzed clinically important cardiopulmonary function indices and other secondary preventive variables by comparing CBCR versus HBCR. There are no published data showing significant differences between outcome variables in CBCR and HBCR programs overall. Furthermore, no studies have compared cardiopulmonary exercise (CPX) and laboratory test results simultaneously. The knowledge gap herein is worthy of focused investigation. Our study aimed to verify significant differences in CPX and laboratory data's clinical measurements by comparing CBCR with HBCR, between 1- and 6-month following respective CR programs.

## 2 Methods

### 2.1 Participants

The study protocol was reviewed and approved by the Ethic's Committee of Myogji Hospital (IRB# MJH2020-05-010). A convenient sample of 121 IHD survivors (mean age = 57.5 ± 11.1; 19 women), previously evaluated at our CR center, were recruited. All patients underwent indicated procedures—percutaneous coronary intervention or coronary artery bypass graft—during their inpatient course in cardiology. Our inclusion criteria were as follows: IHD survivors, participants of CR, all CR patients treated from November 2018 to April 2020. The exclusion criteria were: severe heart failure, unstable angina, uncontrolled arrhythmia, history of major psychiatric illness, other significant noncardiac-related comorbidity, precluding the ability to exercise on the treadmill, readmission for subsequent acute myocardial infarction with previous intervention received. These criteria are based on the CHARMS randomized trial by Dalal in 2007 of participants who underwent prescribed “home” or “center”-based CR following IHD.^[16]^

### 2.2 Design and selection procedures

The study aimed to compare clinical outcomes of CPX indices and laboratory data from the 2 interventional approaches, CBCR versus HBCR programs. The investigation is a retrospective, nonrandomized, nonblinded controlled study design by analyzing patients’ medical records at our institution upon referral for CR from other departments in our medical center. CR specialists—physicians and nurses—interviewed patients to determine their suitability for CR. Patients and their family members were educated on cardiac disease risk factors, emphasizing the importance of lifestyle modification to achieve beneficial results.

**Figure 1:**
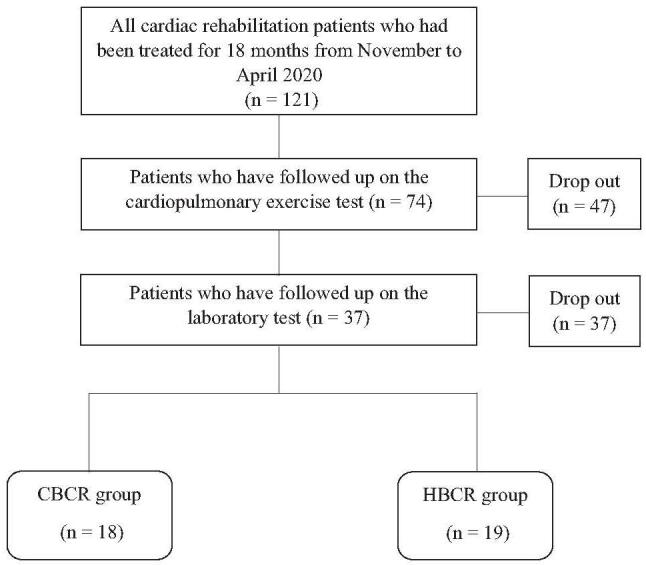
Flow diagram.

The sample size was determined to yield a moderate effect size with a power of 0.80. Among the initial recruits having met eligibility criteria, 47 were eliminated following initial CPX evaluation performed at 1- and 6-month status post CR referral. Laboratories were drawn at 1- and 6-month after enrollment. Thereafter, 37 patients were further eliminated for various reasons based on eligibility criteria, including losses to follow-up. The final study population of 37 was selected for full participation in the study. Participants were allocated to either CBCR or HBCR groups. This was determined according to subjective preferences based on residential location and current lifestyle. The same guidelines (ACSM-2006) were applied to both groups to prescribe the exercise program.^[17]^ The selection process is summarized in Figure 1.

### 2.3 Cardiopulmonary exercise test

The validity and reliability of CPX testing have been substantiated by Nieman et al in 2013 and are outlined in their work assessing energy metabolism during aerobic exercise in healthy subjects.^[18]^ The CPX test was completed in our study following the American College of Cardiology/American Heart Association guidelines.^[19]^ The instrumentation for testing consisted of a Treadmill and Recumbent system (Appendix 1).

### 2.4 Laboratory data measurements

Labs were drawn by diagnostic laboratory and analyzed by an automated chemistry analyzer (Appendix 2); these included high-density lipoprotein (HDL), low-density lipoprotein (LDL), total cholesterol, and creatine (Cr). The ejection fraction (EF) was measured by a cardiologist using portable ultrasound machines at our institution (Appendix 3). The resultant data were uploaded onto the EMR system.

### 2.5 Intervention

The initial discharge planning incorporated an orientation program involving a health education session for all patients regarding their underlying conditions.

Risk factor modifications and adherence to the prescribed program with their benefits were emphasized to enhance attendance and compliance rates.

The initial evaluation involved CPX and laboratory testing regardless of preferred group selection. The prescribed exercise intensity was calculated based on the heart rate reserve achieved during the preliminary graded exercise testing and the rating of perceived exertion (RPE) based on ACSM-2006 guidelines.^[17]^ The applied intensity was based on a target heart rate range using the Karvonen formula. ACSM guidelines also recommend that during exercise, patients should: pay close attention to the difficulty level of the work rate, and this should subjectively reflect the total amount of exertion and fatigue, combining all sense of physical stress effort and fatigue.^[17]^ Guidelines suggest that light-to-moderate intensity (RPE of 11–14) is suitable for cardiac patients and that it is essential to use standardized instruction to minimize misinterpretation of RPE.

### 2.6 Center-based cardiac rehabilitation

Participants in the CBCR group attended outpatient sessions once or twice weekly for 18 to 24 weeks. These sessions included providing feedback on their progress, condition, and education on ongoing lifestyle habits. The outpatient CBCR program's initial stage involved reviewing medical history, diagnosis, medications, lab results, pain symptoms, and pertinent risk factors. We used the cardiac rehab center's instrumentation system (Appendix 4); participants were reeducated on the exercise program's method, purpose, precautions, and other procedure-relevant details.

### 2.7 Home-based cardiac rehabilitation

The home program, primarily focused on brisk walking, is suggested as the preferred activity sufficient to increase aerobic capacity in healthy sedentary and cardiac patients.^[19]^ Healthy lifestyle education with pertinent information on the prescribed home program is reiterated at discharge. Participants were advised to adhere to the specified home program for 12 weeks before returning for the follow-up. The intensity level was adapted to the speed at which the participant previously achieved on the treadmill during the inpatient training.

Participants were advised to contact the center for necessary guidance during home participation. Their exercise log was reviewed every 15 days. When any subjective concerns were reported, trained physiotherapists provided detailed instructions, particularly concerning signs and symptoms during the performance. When necessary, criteria for termination of activity were also explained as per ASCM Guidelines.^[17]^

### 2.8 Statistical analysis

Chi-square test and independent *t* test were used to compare participants’ baseline characteristics, and descriptive statistics included the means with standard deviations.

A “Groups” (CBCR and HBCR) × “Times” (1- and 6-month) repeated measure by ANOVA was used to determine the statistical difference in CPX variables (VO2/kg, metabolic equivalents [METs], minute ventilation/carbon dioxide production slope [VE/VCO2 slope], breathing reserve [BR], VT, heart rate recovery [HRR], oxygen consumption per heart rate [VO2/HR]). Group × Time's significant interaction would indicate that the observed change between 1- and 6-month intervals is significantly different between CBCR and HBCR groups. Tukey post-hoc test was performed if the interaction and main effects were observed. Laboratory data (HDL, LDL, total cholesterol, Cr, EF) at both time-intervals for the groups were included in the comparative analysis, and statistical significance was set at *P* < .05. Statistical Package for the Social Science, Windows v25.0, was used for all statistical analyses.

## 3 Results

All participants who completed CPX and laboratory tests were included in the analysis. Data were acquired after admission and following any necessary cardiac procedures before discharge. Chi-square and independent *t* tests did not show significant differences in the baseline demographic and clinical characteristics between the 2 groups (Table 1).

**Table 1:** Demographic and clinical characteristics (n = 37).

Parameters	CBCR (n = 18)	HBCR (n = 19)	*P*
Gender (male/female)	13/5	18/1	.066
Age (yrs)	56.8 ± 10.2	55.3 ± 8.1	.683
BMI (kg/m^2^)	26.3 ± 5.3	24.7 ± 3.0	.195
Past history			
Hypertension	7 (39%)	8 (42%)	.847
Diabetes mellitus	6 (33%)	5 (26%)	.652
Smoking	8 (44%)	11 (58%)	.427
Alcohol	6 (33%)	9 (47%)	.399
Acute coronary syndrome			
STEMI	11 (61%)	11 (58%)	.847
NSTEMI	5 (28%)	4 (21%)	.645
HF	1 (6%)	1 (5%)	.970
Angina pectoris	1 (6%)	3 (16%)	.330
Major invasive management			
PTCA	16 (89%)	17 (89%)	.956
Laboratory data			
HDL (mg/dL)	47.6 ± 13.3	44.4 ± .5.7	.495
LDL (mg/dL)	127.5 ± 37.2	125.3 ± 32.6	.696
Total (mg/dL)	199.1 ± 46.7	193.7 ± 47.7	.737
Creatinine (mg/dL)	0.9 ± 0.2	0.9 ± 0.3	.184
Ejection fraction (%)	40.7 ± 9.5	47.8 ± 6.3	.135

On pre–post treatment comparison, both groups showed significant improvement in oxygen consumption per kilogram (VO2/kg) (*P* < .001), METs (*P* < .001), VE/VCO2 slope (*P* = .023), BR (*P* = .003), tidal volume (VT) (*P* = .013), HRR (*P* = .001), VO2/HR (*P* = .031), LDL (*P* < .001), LDL/HDL ratio (*P* < .001), total cholesterol (*P* < .001), EF (*P* = .005) respectively. In addition, VO2/kg, METs, and EF improved in the CBCR group compared with the HBCR group on between groups analysis (*P* < .05) (Tables 2 and 3).

**Table 2:** CPX comparison between CBCR and HBCR groups.

	CBCR		HBCR		*P* value		
	1 mo later	6 mo later	1 mo later	6 mo later	Time effect	Time × Group	Between groups
VO_2_/kg^¶^	18.4 ± 5.1	22.1 ± 5.7	22.2 ± 4.4	25.3 ± 6.2	.000^††^	.482	.047^∗∗^
METs^‡^	5.1 ± 1.7	6.3 ± 1.6	6.4 ± 1.2	7.2 ± 1.7	.000^††^	.286	.033^∗∗^
VE/VCO_2_ slope^§^	34.4 ± 8.4	30.9 ± 4.0	36.9 ± 19.2	29.0 ± 5.4	.023^∗∗^	.374	.922
BR^∗^	57.0 ± 15.2	52.2 ± 10.4	56.1 ± 8.5	50.0 ± 11.8	.003^††^	.705	.657
VT^#^	1.5 ± 0.6	1.7 ± 0.6	1.8 ± 0.4	1.9 ± 0.4	.013^∗∗^	.259	.109
HRR^†^	17.9 ± 11.1	24.7 ± 10.4	21.0 ± 9.5	24.9 ± 9.8	.001^††^	.368	.596
VO_2_/HR^||^	10.6 ± 3.1	11.5 ± 2.9	12.9 ± 3.1	13.0 ± 2.9	.031^∗∗^	.159	.199

**Table 3:** Laboratory data comparison between CBCR and HBCR group.

	CBCR		HBCR		*P* value		
	1 mo later	6 mo later	1 mo later	6 mo later	Time effect	Time × Group	Between groups
HDL^‡^	46.6 ± 12.3	41.4 ± 8.7	43.5 ± 8.3	41.6 ± 7.4	.318	.747	.785
LDL^§^	126.7 ± 41.2	65.4 ± 26.6	121.3 ± 42.0	56.1 ± 27.6	.000^#^	.209	.930
LDL/HDL ratio	2.8 ± 1.0	1.6 ± .8	2.8 ± 1.0	1.3 ± .5	.000^#^	.348	.583
Total^||^	197.1 ± 47.7	123.2 ± 35.9	191.7 ± 49.7	113.1 ± 31.7	.000^#^	.234	.909
Creatinine^∗^	.9 ± .3	1.0 ± .36	1.0 ± .4	1.0 ± .4	.349	.448	.717
Ejection fraction^†^	42.7 ± 10.8	49.7 ± 7.8	50.8 ± 8.5	52.5 ± 6.0	.005^#^	.054	.028^¶^

## 4 Discussion

The clinical markers’ importance by evaluating patients with underlying cardiovascular diseases using CPX data is emerging. However, no studies have compared data acquired from CPX and lab testing between the center and home-based groups. Our analysis compared indicators of data acquired by CPX and laboratory evaluation among patients with cardiovascular diseases who underwent CBCR and HBCR. This study confirmed that the time effect of CPX data showed improvement in both groups from 1- to 6-month. Similarly, other variables showing differences between groups indicated a time effect on LDL, LDL/HDL ratio, total cholesterol, and EF.

[VO2/kg] VO2/kg has been reported by several studies in the literature as an independent predictor of long-term survival in CR patients.^[20,21]^

We observed a significant increase in VO2/kg for both groups compared at 1- and 6-month (*P* < .001). The increase in CBCR was marginally greater than HBCR (*P* = .047). Contrary to our observations, Park's study in 2019 found no significant differences in VO2max between groups.^[22]^ A systematic review by Blair et al in 2010 showed improvement in 6-minute walk test, physical daily activity index, functional capacity, and estimated VO2 in both groups with minimal and no difference between groups.^[23]^ These results can often be affected by patients’ motivation and subjective preferences of measurements conceptualized in the study design by trialists. HBCR participants in our study, who had poor motivation for participation, showed a significant difference in oxygen intake compared with the CBCR group.

[METs] METs is an objective measure of the rate at which one expends the energy level relative to their weight while performing a specific activity. One MET is defined as the amount of oxygen consumed during rest and is equivalent to 3.5 mL/kg/min.^[24]^ METs showed a significant increase in both groups with a greater increase among CBCR (*P* < .001, *P* = .033). CBCR participants have the advantage of being monitored during exercise performance; thus, they are more motivated to participate. HBCR participants require a scheduled visit or phone call to assess their interim progress. The different circumstances can easily be a source of discouragement, thereby minimizing willingness or motivation to participate, especially when performing independently at home.

[VE/VCO2] The VE/VCO2 slope is the minute ventilation/carbon dioxide production, indicating ventilatory efficiency. The slope decreased significantly between 1- and 6-month in both groups and without significant difference between groups (*P* = .023, *P* = .922). In the CBCR group, 11 out of 18 participants had a 34 or greater slope at 1 month.

However, only 2 participants maintained this slope at 6-months. In the HBCR group, 9 of 19 participants at 1 month showed a 34 or greater slope, whereas, at 6 months, only 4 participants had maintained this slope.

If the VE/VCO2 slope is greater than 34, it indicates an abnormal response ventilatory inefficiency.^[25]^ In a previous study (Cahalin^[26]^), the VE/VCO2 slope's diagnostic odds ratio was reported to be 5.40 and that there was a stronger association with mortality than with peripheral venous blood gas and exercise duration. And thus, these variables were consequently identified as a robust prognostic predictor.^[27]^

[BR] Breathing reserve significantly increased from 1- to 6-month in both groups. BR (%) is defined as [(1−VEmax/VEmax predicted) × 100]. It is expressed as the difference between maximum exercise ventilation and maximal voluntary ventilation and a ventilatory response indicating the maximum breathing capacity during maximal exercise. In healthy men, BR is at least 10% to 40% of maximal voluntary ventilation. In the setting of lung disease, it is characterized by a low BR, whereas in cardiovascular disease, a high BR appears at a limited amount of exercise.^[28]^ Our study showed a significant decrease in BR for both groups (*P* = .003).

[VT] The tidal volume (VT) increased significantly from 1- to 6-month in both groups (*P* = .013). Molino-Lova et al in 2013 reported in their study the results of a 6-minute walk test before and after CR in the elderly.^[29]^ Patients who underwent median sternotomy showed to have an increase in VT with an increase in metabolic demand. In our study, the metabolic demand of the subjects increased due to the 6-month CR program. Since VT has been shown to increase following CR, inspiratory muscle training for enhancing ventilatory efficiency in IHD should be included in CR programs.

[HRR and VO2/HR] Heart rate recovery showed a significant increase from 1- to 6-month in both groups (*P* = .001). HRR is defined as the heart rate, which decreases 1 minute after exercise. As indicated by Cole et al in 1999, a study of coronary heart disease (CHD) among a cohort of elderly patients reported being a strong predictor of mortality.^[30]^ They reported that change in HR after exercise was under the control of parasympathetic tone. In our study, both groups showed a significant increase in HRR (*P* = .001), indicating that parasympathetic regulatory function was improved after CR.

VO2/HR increased significantly between 1- and 6-month in both groups (*P* = .031). VO2/HR (mL/min) is an oxygen pulse and follows the cardiac stroke volume during exercise. This coincides with the EF and shows a significant increase in cardiac output after CR in both groups. There was a significant difference between groups in EF (*P* = .028), but there was no significant difference between groups in VO2/HR (*P* = .199). This can be attributable to the difference in measurement methods between EF (ultrasound image) and VO2/HR (breathing gas analysis).

[HDL, LDL, total cholesterol, and Creatinine] There was no significant change observed for HDL and Cr levels. Cr plays a vital role in the energy metabolism of heart muscles. It has been reported that the Cr level of cardiac muscle decreases in patients with congestive heart failure.^[31]^ However, Cr levels were within the normal range at 1- and 6-month for both groups without significance. And thus, the effects of CR on renal function are inconclusive based on our observation.

In a study by Streja in 1979, 32 middle-aged men with CHD had no significant LDL changes with an increase in HDL levels after 13 weeks of moderate exercise program, predicting an increase in HDL.^[32]^ Contrary to their prediction, there was no significant difference in HDL for both groups. Our study was conducted during the acute and subacute phases for IHD patients, resulting in a subjectively insufficient amount of exercise to impact underlying HDL levels.

Lavie's study in 1994 showed improvement in HDL, LDL, LDL/HDL ratios, total cholesterol, triglycerides, body mass index, percent body fat, and METs following CR.^[33]^ Similarly, we observed in both groups a significant decrease in LDL, LDL/HDL ratios (*P* < .001, *P* < .001), and total cholesterol from 1- to 6-month (*P* < .0001). In a study by Natarajan et al in 2003, LDL/HDL ratio showed a better predictor of CHD risk reduction than HDL or LDL changes independently.^[34]^ Therefore, both CBCR and HBCR are sufficient to reduce the risk of CHD. These results collaboratively substantiate reports of decreased CHD and atherosclerosis risks in association with reduced LDL, LDL/HDL ratio, and total cholesterol levels.^[35]^

[EF] There was a time effect on EF with a significant increase for both groups (*P* = .005). CBCR showed a greater increase with significance than HBCR (*P* = .028). In a study by Haddadzadeh in 2011, the changes in various indicators following a 12-week exercise program were assessed between CBCR, control group (nothing) (*P* = .003), and HBCR, control group (nothing) (*P* = .04). The resultant changes showed to be significant; however, there was no significant difference in the amount of EF change between CBCR and HBCR (*P* = 1.0).^[36]^ Haddadzadeh's study did not verify whether there was a difference in the EF from baseline. Although a significant difference from baseline EF was unobserved in our study, we confirmed the difference between CBCR and HBCR (*P* = .135). We observed a difference in VO2/kg between the CBCR and HBCR groups, and the change in EF supports this result.

Both CBCR and HBCR are ideal options for improving baseline heart and respiratory functions and lowering the risk of CHD. However, during the acute and subacute periods from 1- to 6-month after fully completing a prescribed CR program of exercises, the CBCR option showed to be more effective than HBCR in yielding improved cardiac output.

Similar to clinical trials for other rehabilitation disciplines programs, randomization was a challenge for this study comparing CBCR to HBCR. Despite the retrospective study design, strict selection criteria yielded many dropouts based on ineligibility per protocol. And thus, the resulting small sample size is another limitation in the study.

## 5 Conclusion

The study confirmed that “time” effects of CPX improved cardiopulmonary function in both groups during the acute and subacute phases of recovery (1- and 6-month period) following CR. CBCR showed greater improvement than HBCR, in cardiac output with significance, in VO2/kg, METs, and EF on between groups analysis (*P* < .05). The results provide further evidence on CR's benefits toward efficient recovery of cardiac function among survivors of cardiovascular disease.

## Acknowledgment

The authors acknowledge Dr. Yong Gyun Kim for his valuable support, input, and guidance in this manuscript.

## Author contributions

**Conceptualization:** Jeong Jae Lee.

**Data curation:** Jeong Jae Lee, Jun Young Ko.

**Investigation:** Jeong Jae Lee.

**Methodology:** Jun Young Ko.

**Project administration:** Seungbok Lee.

**Resources:** Jeong Jae Lee, Jun Young Ko.

**Software:** Jeong Jae Lee.

**Supervision:** Seungbok Lee.

**Validation:** Seungbok Lee.

**Writing – original draft:** Jeong Jae Lee.

**Writing – review & editing:** Jun Young Ko, Seungbok Lee.

## Supplementary Material

Supplemental Digital Content

## Supplementary Material

Supplemental Digital Content

## Supplementary Material

Supplemental Digital Content

## Supplementary Material

Supplemental Digital Content

## References

[1] Francis T Kabboul N Rac V. The effect of cardiac rehabilitation on health-related quality of life in patients with coronary artery disease: a meta-analysis. Can J Cardiol 2019;35:352–64. 30825955 10.1016/j.cjca.2018.11.013

[2] Lavie CJ Thomas RJ Squires RW Allison TG Milani RV. Exercise training and cardiac rehabilitation in primary and secondary prevention of coronary heart disease. Mayo Clin Proc 2009;84:373–83. 19339657 10.1016/S0025-6196(11)60548-X PMC2665984

[3] Jolliffe JA Rees K Taylor RS Thompson D Oldridge N Ebrahim S. Exercise-based rehabilitation for coronary heart disease. Cochrane Database Syst Rev 2001;CD001800. 11279730 10.1002/14651858.CD001800

[4] Heran BS Chen JM Ebrahim S. Exercise-based cardiac rehabilitation for coronary heart disease. Cochrane Database Syst Rev 2011;CD001800. 21735386 10.1002/14651858.CD001800.pub2 PMC4229995

[5] Anderson L Taylor RS. Cochrane Heart Group. Cardiac rehabilitation for people with heart disease: an overview of Cochrane systematic reviews. Cochrane Database Syst Rev 2014;CD011273. 10.1002/14651858.CD011273.pub2 PMC7087435 25503364

[6] Anderson L Sharp GA Norton RJ. Home-based versus centre-based cardiac rehabilitation. Cochrane Database of Systematic Reviews. 2017;6:CD007130. 10.1002/14651858.CD007130.pub4 PMC6481471 28665511

[7] Kabboul NN Tomlinson G Francis TA. Comparative effectiveness of the core components of cardiac rehabilitation on mortality and morbidity: a systematic review and network meta-analysis. J Clin Med 2018;7:514. 10.3390/jcm7120514 PMC6306907 30518047

[8] Santiago de Araújo Pio C Beckie TM Varnfield M. Promoting patient utilization of outpatient cardiac rehabilitation: a joint International Council and Canadian Association of Cardiovascular Prevention and Rehabilitation position statement. Int J Cardiol 2020;298:01–7. 10.1016/j.ijcard.2019.06.064 31405584

[9] Turk-Adawi K Supervia M Lopez-Jimenez F. Cardiac rehabilitation availability and density around the globe. EClinicalMedicine 2019;13:31–45. 31517261 10.1016/j.eclinm.2019.06.007 PMC6737209

[10] Cortés O Arthur HM. Determinants of referral to cardiac rehabilitation programs in patients with coronary artery disease: a systematic review. Am Heart J 2006;151:249–56. 16442885 10.1016/j.ahj.2005.03.034

[11] Thomas RJ King M Lui K. AACVPR/ACC/AHA 2007 Performance Measures on Cardiac Rehabilitation for Referral to and Delivery of Cardiac Rehabilitation/Secondary Prevention Services: Endorsed by the American College of Chest Physicians, American College of Sports Medicine, American Physical Therapy Association, Canadian Association of Cardiac Rehabilitation, European Association for Cardiovascular Prevention and Rehabilitation, Inter-American Heart Foundation, National Association of Clinical Nurse Specialists, Preventive Cardiovascular Nurses Association, and the Society of Thoracic Surgeons. JACC CardioOncol 2007;50:1400–33. 10.1016/j.jacc.2007.04.033 17903645

[12] Shanmugasegaram S Gagliese L Oh P. Psychometric validation of the Cardiac Rehabilitation Barriers Scale. Clin Rehabil 2012;26:152–64. 21937522 10.1177/0269215511410579 PMC3351783

[13] Santiago de Araújo Pio C Chaves GS Davies P Taylor RS Grace SL. Interventions to promote patient utilisation of cardiac rehabilitation. The Cochrane Database of Systematic Reviews. J Clin Med 2019;8:189. 10.1002/14651858.CD007131.pub4 PMC6360920 30706942

[14] Santiago de Araújo Pio C Gagliardi A Suskin N Ahmad F Grace SL. Implementing recommendations for inpatient healthcare provider encouragement of cardiac rehabilitation participation: development and evaluation of an online course. BMC Health Serv Res 2020;1:768. 10.1186/s12913-020-05619-2 PMC7439558 32819388

[15] Scalvini S Zanelli E Comini L. Home-based versus in-hospital cardiac rehabilitation after cardiac surgery: a nonrandomized controlled study. Phys Ther 2013;93:1073–83. 23599353 10.2522/ptj.20120212

[16] Dalal HM Evans PH Campbell JL. Home-based versus hospital-based rehabilitation after myocardial infarction: a randomized trial with preference arms – Cornwall Heart Attack Rehabilitation Management Study (CHARMS). Int J Cardiol 2007;119:202–11. 17196274 10.1016/j.ijcard.2006.11.018

[17] ACSM, 8th ed. Philadelphia, PA: Lippincott, Williams and Wilkins. American College of Sports Medicine (ACSM) – Guidelines for exercise testing and prescription; 2006.

[18] Nieman DC Austin MD Dew D Utter AC. Validity of COSMED's Quark CPET mixing chamber system in evaluating energy metabolism during aerobic exercise in healthy male adults. Res Sports Med 2013;21:136–45. 23541100 10.1080/15438627.2012.757227

[19] Marchionni N Fattirolli F Fumagalli S. Improved exercise tolerance and quality of life with cardiac rehabilitation of older patients after myocardial infarction: results of a randomized controlled trial. Circulation 2003;107:2201–6. 12707240 10.1161/01.CIR.0000066322.21016.4A

[20] Williams SG Cooke GA Wright DJ. Peak exercise cardiac power output: a direct indicator of cardiac function strongly predictive of prognosis in chronic heart failure. Eur Heart J 2001;22:1496–503. 11482923 10.1053/euhj.2000.2547

[21] Sinagra G Iorio A Marco M. Prognostic value of cardiopulmonary exercise testing in Idiopathic Dilated Cardiomyopathy. Int J Cardiol 2016;223:596–603. 27561166 10.1016/j.ijcard.2016.07.232

[22] Park HK Kim KH Kim JH Song MK Choi IS Han JY. Comparison of obesity related index and exercise capacity between center-based and home-based cardiac rehabilitation programs. Ann Rehabil Med 2019;43:297–304. 31311251 10.5535/arm.2019.43.3.297 PMC6637052

[23] Blair J Corrigall H Angus NJ Thompson DR Leslie S. Home versus hospital-based cardiac rehabilitation: a systematic review. Rural Remote Health 2011;11:1532. 21488706

[24] Ainsworth BE Haskell WL Leon AS. Compendium of physical activities: classification of energy costs of human physical activities. Med Sci Sports Exerc 1993;25:71–80. 8292105 10.1249/00005768-199301000-00011

[25] Corrà U Mezzani A Bosimini E Giannuzzi P. Cardiopulmonary exercise testing and prognosis in chronic heart failure: a prognosticating algorithm for the individual patient. Chest 2004;126:942–50. 15364777 10.1378/chest.126.3.942

[26] Cahalin LP Chase P Arena R. A meta-analysis of the prognostic significance of cardiopulmonary exercise testing in patients with heart failure. Heart Fail Rev 2013;18:79–94. 22733204 10.1007/s10741-012-9332-0 PMC7245616

[27] Arena R Myers J Aslam SS Varughese EB Peberdy MA. Peak VO_2_ and VE/VCO_2_ slope in patients with heart failure: a prognostic comparison. Am Heart J 2004;147:354–60. 14760336 10.1016/j.ahj.2003.07.014

[28] Toma N Bicescu G Enache R Dragoi R Cinteza M. Cardiopulmonary exercise testing in differential diagnosis of dyspnea. Maedica (Bucur) 2010;5:214–8. 21977155 PMC3177547

[29] Molino-Lova R Pasquini G Vannetti F. Ventilatory strategies in the six-minute walk test in older patients receiving a three-week rehabilitation programme after cardiac surgery through median sternotomy. J Rehabil Med 2013;45:504–9. 23468060 10.2340/16501977-1126

[30] Cole CR Blackstone EH Pashkow FJ Snader CE Lauer MS. Heart-rate recovery immediately after exercise as a predictor of mortality. N Engl J Med 1999;341:1351–7. 10536127 10.1056/NEJM199910283411804

[31] Kuethe F Krack A Richartz BM Figulla HR. Creatine supplementation improves muscle strength in patients with congestive heart failure. Pharmazie 2006;61:218–22. 16599263

[32] Streja D Mymin D. Moderate exercise and high-density lipoprotein-cholesterol observations during a cardiac rehabilitation program. JAMA 1979;242:2190–2. 226733

[33] Lavie CJ Milani RV. Effects of cardiac rehabilitation and exercise training on low-density lipoprotein cholesterol in patients with hypertriglyceridemia and coronary artery disease. Am J Cardiol 1994;74:1192–5. 7977088 10.1016/0002-9149(94)90546-0

[34] Natarajan S Glick H Criqui M Horowitz D Lipsitz SR Kinosian B. Cholesterol measures to identify and treat individuals at risk for coronary heart disease. Am J Prev Med 2003;25:50–7. 12818310 10.1016/s0749-3797(03)00092-8

[35] William BK. Range of serum cholesterol values in the population developing coronary artery disease. Am J Cardiol 1995;76:69C–77C. 10.1016/s0002-9149(99)80474-3 7572691

[36] Haddadzadeh MH Maiya AG Padmakumar R Shad B Mirbolouk F. Effect of exercise-based cardiac rehabilitation on ejection fraction in coronary artery disease patients: a randomized controlled trial. Heart Views 2011;12:51–7. 22121461 10.4103/1995-705X.86013 PMC3221192

