# Supplementary material for: An observational study substantiating the statistical significance of cardiopulmonary exercise with laboratory tests during the acute and subacute phases of center and home-based cardiac rehabilitation
Source: Medicine (Baltimore). 2021 Aug 6;100(31):e26861. doi: 10.1097/MD.0000000000026861 (PMC8341314; doi:10.1097/MD.0000000000026861)
Supplement: Supplemental Digital Content [file medi-100-e26861-s001.docx]

Appendix 1.

Treadmill and Recumbent test system:

ECG monitoring system (CASE, GE Medical System Information Technologies, Inc., Milwaukee, USA).

A Stress test monitor (Tango M2, SunTech Medical, North Carolina, USA).

Metabolic gas analyzer (Quark CPET, COSMED The Metabolic Company, Roma, Italia).

Treadmill (T150, COSMED The Metabolic Company, Roma, Italia ).

Recumbent (Lode BV Medical Technology, Groningen, Netherlands).
