# Supplementary material for: An observational study substantiating the statistical significance of cardiopulmonary exercise with laboratory tests during the acute and subacute phases of center and home-based cardiac rehabilitation
Source: Medicine (Baltimore). 2021 Aug 6;100(31):e26861. doi: 10.1097/MD.0000000000026861 (PMC8341314; doi:10.1097/MD.0000000000026861)
Supplement: Supplemental Digital Content [file medi-100-e26861-s002.docx]

Appendix 2.

Clinical chemistry analyzer (ADVIA Chemistry XPT System, SIEMENS, Berlin, Germany).
