# Supplementary material for: An observational study substantiating the statistical significance of cardiopulmonary exercise with laboratory tests during the acute and subacute phases of center and home-based cardiac rehabilitation
Source: Medicine (Baltimore). 2021 Aug 6;100(31):e26861. doi: 10.1097/MD.0000000000026861 (PMC8341314; doi:10.1097/MD.0000000000026861)
Supplement: Supplemental Digital Content [file medi-100-e26861-s003.docx]

Appendix 3.

CX50 Philips Eindhoven Netherlands.

EPIQ CVx Philips Eindhoven Netherlands.

IE-33DS Philips Eindhoven, Netherlands.

VIVID E9 GE Healthcare Milwaukee, USA.

VIVID i GE Healthcare Milwaukee, USA.

VIVID Q GE Healthcare Milwaukee, USA.
