# Supplementary material for: An observational study substantiating the statistical significance of cardiopulmonary exercise with laboratory tests during the acute and subacute phases of center and home-based cardiac rehabilitation
Source: Medicine (Baltimore). 2021 Aug 6;100(31):e26861. doi: 10.1097/MD.0000000000026861 (PMC8341314; doi:10.1097/MD.0000000000026861)
Supplement: Supplemental Digital Content [file medi-100-e26861-s004.docx]

Appendix 4.

Rehabilitation exercise monitoring system ((Life Scope G3 [4ch]GZ-130P, Nihon Kohden, Tokyo, Japan).

Wearable rehabilitation bicycle (CRB-450, MOTUS; Paju, South Korea).

Wearable rehabilitation treadmill (T7M, MOTUS; Paju, South Korea).
